# Potential Probiotic Properties of Exopolysaccharide-Producing *Lacticaseibacillus paracasei* EPS DA-BACS and Prebiotic Activity of Its Exopolysaccharide

**DOI:** 10.3390/microorganisms10122431

**Published:** 2022-12-08

**Authors:** Min-Gyu Lee, Huijin Joeng, Jaein Shin, Suin Kim, Chaeeun Lee, Youngbo Song, Byung-Hoo Lee, Hyoung-Geun Park, Tae-Ho Lee, Hai-Hua Jiang, Young-Sun Han, Bong-Gyeong Lee, Ho-Jin Lee, Min-Ju Park, Yun-Ju Jun, Young-Seo Park

**Affiliations:** 1 Department of Food Science and Biotechnology, Gachon University, Seongnam 13120, Republic of Korea; 2 Research Laboratory, Dong-A Pharmaceutical Co., Ltd., Yongin 17073, Republic of Korea

**Keywords:** exopolysaccharide, *Lacticaseibacillus paracasei*, probiotics, lactic acid bacteria, anti-inflammatory activity, gastrointestinal tolerance, prebiotics, antimicrobial activity

## Abstract

Exopolysaccharide (EPS)-producing *Lacticaseibacillus paracasei* EPS DA-BACS was isolated from healthy human feces and its probiotic properties, as well as the structure and prebiotic activity of the EPS from this strain were examined. EPS from *L. paracasei* EPS DA-BACS had a ropy phenotype, which is known to have potential health benefits and is identified as loosely cell-bounded glucomannan-type EPS with a molecular size of 3.7 × 10^6^ Da. EPS promoted the acid tolerance of *L. paracasei* EPS DA-BACS and provided cells with tolerance to gastrointestinal stress. The purified EPS showed growth inhibitory activity against *Clostridium difficile. L. paracasei* EPS DA-BACS cells completely inhibited the growth of *Bacillus subtilis*, *Pseudomonas aeruginosa*, and *Aspergillus brasiliensis*, as well as showed high growth inhibitory activity against *Staphylococcus aureus* and *Escherichia coli*. Treatment of lipopolysaccharide-stimulated RAW 264.7 cells with heat-killed *L. paracasei* EPS DA-BACS cells led to a decrease in the production of nitric oxide, indicating the anti-inflammatory activity of *L. paracasei* EPS DA-BACS. Purified EPS promoted the growth of *Lactobacillus gasseri, Bifidobacterium bifidum, B. animalis*, and *B. faecale* which showed high prebiotic activity. *L. paracasei* EPS DA-BACS harbors no antibiotic resistance genes or virulence factors. Therefore, *L. paracasei* EPS DA-BACS exhibits anti-inflammatory and antimicrobial activities with high gut adhesion ability and gastrointestinal tolerance and can be used as a potential probiotic.

## 1. Introduction

Lactic acid bacteria (LAB), especially the genus *Lactobacillus*, are known to be beneficial for human health and thus, are usually considered probiotics. Probiotics are live microorganisms that, when administered in adequate amounts, confer a health benefit on the host [1]. Probiotics can benefit human health by inhibiting the growth of intestinal pathogens, promoting the growth of beneficial microorganisms, reducing serum cholesterol concentration, and exerting antioxidant activity. These effects may be caused by the microorganisms themselves or their metabolites such as bacteriocins, exopolysaccharides, and organic acids [2].

Many studies have reported the benefits of *Lacticaseibacillus paracasei* [3,4]. These include their antioxidant, anti-inflammatory, anti-obesity, anti-pathogenic, microbiota modulatory, antimicrobial, and gut microbiota modulatory activities. These effects are attributed to the effector molecules. Exopolysaccharides (EPSs) produced by *L. paracasei* are regarded as postbiotics and secreted into the environment in the form of slime or are attached to the cell surface in the form of a capsule [5].

EPS can be used as a replacement for antibiotics. EPS is not used as an energy source but as a protective mechanism against external environmental factors, such as toxic metals, host innate immune factors, phage attacks, and desiccation [6]. Furthermore, the EPS layer in bacterial cells is thought to be involved in the protection against adverse environmental conditions of the gastrointestinal tract (GIT), including low pH, bile salts, and gastric and pancreatic enzymes [6,7].

EPS produced by lactic acid bacteria has health-promoting properties. It can stimulate the growth of beneficial bacteria, inhibit bacterial adhesion to the intestinal epithelium, increase intestinal barrier integrity by up-regulating tight junctions, and influence the immune system through direct and indirect interactions with Toll-like receptors [8]. EPS can be largely classified into homopolysaccharides and heteropolysaccharides depending on the monomer composition and mechanisms. Homopolysaccharides are composed of only one type of monosaccharides such as fructose and glucose. Levan and inulin are fructans produced by *Streptococcus salivarius* and *S. mutans*. As glucans, dextran is produced by *Leuconostoc mesenteroides* subsp. *mesenteroides* and *Leu. mesenteroides* subsp. *dextranicum*, alterman is produced by *Leu. Mesenteroides*, and mutan is produced by *S. mutans* and *S. sobrinus* [9,10]. Heteropolysaccharides mainly consist of two or more monosaccharides such as galactose, fructose, and rhamnose. In some cases, substances that are not sugars, such as *N*-acetyl-aminosugar, phosphate acetyl, or glycerol, can be present in heteropolysaccharides. It is usually produced by mesophilic lactic acid bacteria and thermophilic lactic acid bacteria but shows lower productivity than homopolysaccharides [9,11]. EPSs from *Bifidobacteria*, *Lactobacillus delbrueckii* subsp. *bulgalicus*, and *Lactobacillus helveticus* var. *jugurti* have been reported to have anticancer effects [12]. EPS produced by streptococci has immunological, anti-ulcer, and cholesterol-lowering activities [13]. Probiotics and their metabolites such as EPS have been reported for their ability to reduce serum cholesterol levels. Therefore, EPS can be used as a food additive owing to its wide bioactivity spectrum and usage, which may have potential applications in the pharmaceutical and food industries. A recent study reported that EPS produced by *L. paracasei* M7 has excellent functional characteristics, such as antioxidant activity, hydroxyl radical scavenging activity, and antibiofilm potential against several human pathogens [14]. In the immunomodulatory potential of *L. paracasei*, research has shown that *L. paracasei* subsp. *paracasei* NTU 101 EPS can induce interleukin-6, interleukin-1β, and tumor necrosis factor-α production [15].

This study was designed to identify *L. paracasei* EPS DA-BACS isolated from human feces and to evaluate the stability of *L. paracasei* in gastrointestinal conditions, as well as its anti-inflammatory and antimicrobial activities against pathogens. The structure of EPS produced by *L. paracasei* was also analyzed and its prebiotic activity was examined.

## 2. Materials and Methods

### 2.1. Bacterial Strains and Growth Condition

Twelve *L. paracasei* strains, namely, KCTC 3169, KCTC 13090, KCTC 3165, KCTC 3189, KCTC 5546, KCTC 3510, KCTC 5058, KCTC 3074, KCCM 40995, KCCM 42830, KCCM 32822, and KCCM 41276 were purchased from the Korean Culture Collection for Type Cultures (Daejeon, Korea) and Korean Culture Center of Microorganisms (Seoul, Korea). *L. paracasei* KB28, *L. paracasei* DLP 1354, *Lactobacillus gasseri* DLP1202, *Bifidiobacterium bifidum* DLP1224, *B. animalis* DLP1267, *B. faecale* DLP1470, *Pseudomonas aeruginosa* ATCC 9027, *Bacillus subtilis* ATCC 6633, *Escherichia coli* ATCC 8739, *Staphylococcus aureus* ATCC 6538, and *Clostridium difficile*, *Aspergillus brasiliensis* ATCC 16404 were provided by DONG-A PHARM (Seoul, Korea). The strains were grown in De Man, Rogosa, and Sharp (MRS, Oxoid, Hampshire, UK) at 37 °C for 24 h under anaerobic condition. The strains were subcultured weekly on MRS agar and stored at 4 °C.

### 2.2. Isolation of EPS-Producing LAB

#### 2.2.1. Isolation of LAB Strains

Feces from healthy Korean volunteers were collected, ten-fold serially diluted with 0.85% (*w/v*) sodium chloride, and 100 µL of diluted sample was spread onto an LBS-selective agar plate (Lactobacillus selective media, MB cell, Seoul, Korea) and incubated at 37 °C for 48 h under anaerobic conditions. The study design was reviewed by the Public Institutional Review Board assigned by the Ministry of Health and Welfare (IRB approval number: P01-202102-33-002).

#### 2.2.2. Isolation of Ropy EPS-Producing LAB Strains

Each LAB strain isolated in this study was cultured on an MRS agar plate containing 10% (*w*/*v*) sucrose and incubated at 37 °C for 72 h under anaerobic conditions. After incubation, the viscous substance of a single colony was identified using a sterilized loop, and the increased length of the colony ropy was measured in millimeters.

### 2.3. Identification of Lactic Acid Bacteria

#### 2.3.1. Identification of *L. paracasei* EPS DA-BACS

Isolation of genomic DNA from *Lacticaseibacillus* strains, DNA amplification by polymerase chain reaction, and nucleotide sequence analysis of the 16S rRNA gene was carried out by Macrogen Inc. (Seoul, Korea). For phylogenetic analysis, CLUSTAL software (v.2.0) was used for multiple sequence alignment and Molecular Evolutionary Genetics Analysis ver. 7 program (MEGA7) was used to prepare phylogenetic trees using the neighbor-joining method.

#### 2.3.2. Biochemical Characteristics of *L. paracasei* EPS DA-BACS

Carbohydrate utilization was evaluated using an API 50 CHL kit (API system, bioMérieux, Montalieu-Vercieu, France). Five colonies of the strain on MRS agar plates were inoculated in 5 mL saline (0.85% (*w*/*v*) NaCl) and mixed. Then, 200 µL of the resuspended solution was added to the API 50 CHL medium and mixed. The mixtures were suspended in API 50 CHL strips and mineral oil (bioMérieux) was overlaid to maintain anaerobic conditions. The strips were incubated at 37 °C and examined at 24 and 48 h. The data were interpreted using Apiweb^TM^ (bioMérieux), available at https://apiweb.biomerieux.com/ (accessed on 25 August 2022).

### 2.4. Production of EPS from LAB and Removal of EPS

To produce EPS, a single colony of LAB was inoculated into basal MRS (MRS without glucose) with 2% (*w*/*v*) sucrose and incubated at 35 °C for 24 h under anaerobic conditions. One milliter of the culture was centrifuged at 11,000× *g* for 20 min to remove EPS from LAB and the cell pellet was washed three times with 1 mL of 0.85% (*w*/*v*) NaCl.

### 2.5. Visualization of EPS

#### 2.5.1. Crystal Violet Staining

Crystal violet staining was performed as described by Oleksy and Klewicka [16]. Briefly, *L. paracasei* EPS DA-BACS was cultured in a basal MRS medium containing 2% (*w*/*v*) sucrose at 35 °C for 48 h under anaerobic conditions. The culture was spread on a microscope slide glass and allowed to air-dry. The culture was stained with 1% (*w*/*v*) crystal violet for 2 min, washed with 20% (*w*/*v*) copper sulfate solution, air-dried, and examined under a light microscope.

#### 2.5.2. Measuring the Ropiness of EPS

*L. paracasei* EPS DA-BACS was cultured on MRS agar plates containing 10% (*w*/*v*) sucrose and was incubated at 37 °C for 72 h under anaerobic conditions. After incubation, the viscous substance of a single colony was identified using an inoculating loop, and the length of the colony was measured in millimeters.

#### 2.5.3. Scanning Electron Microscopy of L. paracasei EPS DA-BACS

The morphology of *L. paracasei* EPS DA-BACS was analyzed using a scanning electron microscope (SEM, H-7600, Hitachi, Tokyo, Japan) installed at Eulji University (Seongnam, Korea) as described previously [17].

### 2.6. Production and Purification of EPS

The culture broth of *L. paracasei* EPS DA-BACS was centrifuged at 11,000× *g* for 20 min and the supernatant was obtained. Trichloroacetic acid was added to the supernatant at a final concentration of 5% (*v*/*v*) and incubated overnight at 4 °C to precipitate the proteins. After incubation, the supernatant was centrifuged at 11,000× *g* for 20 min, and the protein-free supernatant was filtered using a 0.45 μm SmartPor^®^-11PVDF syringe filter (Woongki Science Co., Ltd., Seoul, Korea). Ice-cold 100% ethanol was added at twice the volume to the filtered supernatant, incubated at 4 °C overnight, and centrifuged at 11,000× *g* for 20 min to obtain EPS. The collected EPS was dissolved in distilled water and dialyzed against distilled water using Spectra/Por^®^ 4 Dialysis Membrane Standard RC Tubing (12-14 kD molecular weight cut-off; Spectrum Laboratories, Inc., Broadwick Street, Rancho Dominguez, CA, USA) for 24 h. The dialyzed purified EPS was freeze-dried using a freeze dryer (SUNILEYELA Co., Ltd., Seongnam, Republic of Korea).

### 2.7. Analysis of EPS Structure

#### 2.7.1. Analysis of Monosaccharide Composition

Monosaccharide composition of the purified EPS sample from *L. paracasei* EPS DA-BACS was analyzed using a high-performance anion-exchange chromatography (HPAEC) system (Dionex, Sunnyvale, CA, USA) as described previously [17].

#### 2.7.2. Analysis of Molecular Size

Purified EPS from *L. paracasei* EPS DA-BACS was solubilized in deionized water (0.2%, *w*/*v*) and filtered through a 0.45 μm nylon syringe filter (GVS). The prepared sample solution was injected into a high-performance size-exclusion chromatography (HPSEC, Ultimate 3000, Thermo Fisher Scientific, Waltham, MA, USA) equipped with a refractive index detector (RID, Thermo Fisher Scientific) at 50 °C. Chromatographic separation was achieved using Shodex OHpak SB-806 and SB-804 HQ analytical columns (Shodex, Tokyo, Japan). Filtered deionized water (18 mΩ) was used as the column eluent at a 0.4 mL/min flow rate [18]. A regression line was prepared using Shodex P-82 pullulan standards (Showa Denko, Tokyo, Japan).

#### 2.7.3. Methylation Analysis for Linkage Pattern

Derivatization of purified EPS from *L. paracasei* EPS DA-BACS to alditol acetates was performed as previously described [19]. Linkage pattern of purified EPS from *L. paracasei* EPS DA-BACS was analyzed as described previously [17].

### 2.8. Gastrointestinal Tract Tolerance

Acid-, bile-, and pancreatin-tolerances of *L. paracasei* EPS DA-BACS were evaluated as described previously except that treated samples were serially diluted and added to a Petri dish with molten MRS agar [17]. MRS agar plates were incubated at 37 °C for 48 h and acid-, bile-, and pancreatin-tolerances were calculated using Equations (1)–(3), respectively.

(1) $$\;\mathrm{Acid}-\mathrm{tolerance}\left(\%\right)=\frac{\log\left(\mathrm{Viable}\;\mathrm{cell}\;\mathrm{number}\;\mathrm{of}\;\mathrm{sample}\right)}{\log\left(\mathrm{Viable}\;\mathrm{cell}\;\mathrm{number}\;\mathrm{of}\;\mathrm{control}\right)}\times 100$$

(2) $$\mathrm{Bile}-\mathrm{tolerance}\left(\%\right)=\frac{\log\left(\mathrm{Viable}\;\mathrm{cell}\;\mathrm{number}\;\mathrm{of}\;\mathrm{sample}\right)}{\log\left(\mathrm{Viable}\;\mathrm{cell}\;\mathrm{number}\;\mathrm{of}\;\mathrm{control}\right)}\times 100\;$$

(3) $$\mathrm{Pancreatin}-\mathrm{tolerance}\left(\%\right)=\frac{\log\left(\mathrm{Viable}\;\mathrm{cell}\;\mathrm{number}\;\mathrm{of}\;\mathrm{sample}\right)}{\log\left(\mathrm{Viable}\;\mathrm{cell}\;\mathrm{number}\;\mathrm{of}\;\mathrm{control}\right)}\times 100\;$$

### 2.9. Antibacterial and Antifungal Activity of LAB

Antibacterial activity was evaluated as follows: The culture broth of *L. paracasei* EPS DA-BACS was inoculated into MRS broth and incubated at 37 °C for 24 h under anaerobic conditions. The culture broth was centrifuged at 1508× *g* for 10 min and the supernatant was filtered. The filtrate was diluted to 1/10th with Mueller Hinton II medium consisting of 17.5 g casein hydrolysate, 2.0 g beef infusion solids, and 1.5 g starch, per liter (Sigma-Aldrich, St. Louis, MO, USA), and 200 μL was dispensed into a 96-well plate. The indicator strains, *P. aeruginosa*, *Bacillus subtilis*, *E. coli*, and *S. aureus* were cultured in nutrient broth containing 3.0 g beef extract, and 5.0 g peptone, per liter (Becton, Dickinson and Company, Franklin Lakes, NJ, USA). The culture broth of the indicator strains was inoculated in a 96-well plate at a final concentration of 1% (*v*/*v*). The filtrate of the culture supernatant was used as the blank group, and the culture broth of indicator strains grown in MH medium was used as the control group. A 96-well plate was incubated at 37 °C for 24 h and cell growth was measured at 600 nm.

Antifungal activity was evaluated as described by Inglin et al. [20]. Briefly, a 24-well cell plate with 300 μL of MRS agar containing 0.1 M K_2_HPO_4_ in each well was used. A 0.75 μL of LAB culture was inoculated at the center of a well and incubated for 24 h at 37 °C under anaerobic conditions. Then, each well was overlaid with 100 μL of 0.5% Yeast Malt soft agar (Oxoid) containing 0.1 M K_2_HPO_4_ and was inoculated with *A. brasiliensis* at the concentration of 10^3^–10^4^ fungal spores/mL. Yeast Malt soft agar without inoculation was used as the blank, and the well inoculated with 0.75 μL peptone instead of LAB culture was used as the control. The plate was incubated at 35 °C for 48 h and the results were measured visually.

### 2.10. Growth Inhibitory Activity of EPS and Culture Broth of L. paracasei EPS DA-BACS against Clostridium Difficile

*Clostridium difficile* was cultured in tryptic soy broth (TSB) by incubating at 37 °C for 24 h. To determine the growth inhibitory activity of EPS, 1.0 × 10^5^ CFU/mL of *C. difficile* cells were inoculated into 200 µL of TSB in a 96-well plate, and EPS produced by *L. paracasei* EPS DA-BACS was added at a final concentration of 50 mg/mL. To determine the growth inhibitory activity of the culture broth of *L. paracasei* EPS DA-BACS, 1.0 × 10^5^ CFU/mL of *C. difficile* cells were inoculated with 180 µL of TSB in 96-well plates and 20 µL of the culture supernatant of 1.0 × 10^9^ CFU/mL of *L. paracasei* EPS DA-BACS culture was added to it. The plates were incubated at 37 °C under anaerobic conditions and optical density (OD) was measured every hour at 600 nm.

### 2.11. Gut Adhesion Ability Assay

Gut adhesion ability was assayed as described previously and calculated using Equation (4) [17,21].

(4) $$Adhesion\;ability\;\left(\%\right)=\frac{log\left(Adhered\;bacteria\;cell\;number\right)\;}{log\left(Initial\;bacterial\;cell\;number\right)}\times 100$$

### 2.12. Cell Viability Assay

The evaluation of cell viability of RAW 264.7 macrophage was commissioned by GeoVista (Hwaseong, Korea) and carried out as described previously [17].

### 2.13. Anti-Inflammatory Activity Assay

Anti-inflammatory activity assay was carried out as described previously with a slight modification [22]. RAW 264.7 cells were inoculated in a 6-well plate at 5 × 10^5^ cells per well and incubated at 37 °C with 5% CO_2_ for 20 h. RAW 264.7 cells were treated with heat-killed *L. paracasei* EPS DA-BACS cells as described in Section 2.12. RAW 264.7 cells treated with 1 μg/mL LPS were used as a positive control. NO secretion was determined using the Griess Reagent System (G2930, Promega, Madison, WI, USA). Dexamethasone was added instead of LAB at a concentration of 1 µM as a positive control The cells were incubated for 2 h, followed by the addition of 1 μg/mL LPS and incubation at 37 °C for 24 h.

### 2.14. Prebiotic Activity of Purified-EPS

To analyze the prebiotic activity of purified EPS, 100 μL of basal MRS broth or TSB broth without glucose, supplemented with 2% (*w*/*v*) EPS, was loaded into a 96-well plate. Cultures of *L. paracasei* EPS DA-BACS, *Lactobacillus gasseri*, *B. bifidum*, *B. animalis*, and *B. faecale* were inoculated at a final concentration of 1% (*v*/*v*). *C. difficile*, *E. coli*, and *Bacteroides fragilis* were inoculated in TSB broth without glucose supplemented with 2% (*w*/*v*) EPS at a final concentration of 1% (*v*/*v*). For comparative experiments, the same experiment was carried out on MRS broth, TSB broth basal MRS broth, or TSB broth without glucose supplemented with 2% (*w*/*v*) inulin as representative prebiotics. The same experiment was carried out using MRS broth without glucose and TSB broth without glucose as a blank and MRS broth with 2%(*w*/*v*) glucose or TSB broth with 2% (*w*/*v*) glucose as a control. The prebiotic index was calculated using Equation (5) according to Hussein et al. [23].

(5) $$\mathrm{Prebiotic}\;\mathrm{index}\;=\frac{\mathrm{Optical}\;\mathrm{density}\;\mathrm{of}\;\mathrm{the}\;\mathrm{probiotic}\;\mathrm{culture}\;\mathrm{at}\;600\;\mathrm{nm}\;}{\mathrm{Optical}\;\mathrm{density}\;\mathrm{of}\;E.\;\mathrm{coli}\;\mathrm{culture}\;\mathrm{at}\;600\;\mathrm{nm}}$$

### 2.15. Whole Genome Sequencing of L. paracasei EPS DA-BACS

Whole-genome sequencing of *L. paracasei* EPS DA-BACS was carried out by CJ Bioscience Inc. (Seoul, Korea) using the Illumina MiSeq platform (Illumina, San Diego, CA, USA). Screening of the antibiotic resistance genes and virulence genes in genomic sequences was performed as described previously [17]. The genome sequence of *L. paracasei* EPS DA-BACS was deposited in NCBI GenBank under the accession number PRJNA880719.

### 2.16. Antibiotics Susceptibility Test

Antibiotic susceptibility was examined using the E-test as described previously [17]. The minimum inhibitory concentrations (MIC) of *L. paracasei* EPS DA-BACS were determined by using the criteria recommended by the European Food Safety Authority (ESFA) guidelines [24].

### 2.17. Statical Analysis

All experiments were carried out in triplicates, and the results were expressed as the mean ± standard deviation (SD). Statistical significance of the data was verified by one-way analysis of variance (ANOVA) using SPSS Statistics 25 (SPSS Inc., Chicago, IL, USA). Duncan’s test and Dunnett’s test were used to determine significant differences between multiple groups at *p* < 0.05.

## 3. Results and Discussion

### 3.1. Isolation and Identification of L. paracasei EPS DA-BACS

To isolate EPS-producing LAB, 261 bacteria were screened in human feces. Of the isolated bacteria, 225 strains of *Lactobacillus* sp., seven strains of *Weissella* sp., and 29 strains of *Pediococcus* sp. were identified. Among these, *Lacticaseibacillus* sp. EPS DA-BACS was selected as the EPS-producing LAB with a highly ropy colony. Analysis of the nucleotide sequence of the 16S rRNA gene revealed that the strain EPS DA-BACS showed 100% homology with *Lacticaseibacillus paracasei* JCM 8130 and was therefore identified as *L. paracasei* by phylogenetic analysis (Supplementary Figure S1). Analysis of the carbohydrate utilization patterns of *L. paracasei* EPS DA-BACS revealed that it could utilize ribose, galactose, glucose, fructose, mannose, sorbose, mannitol, sorbitol, *N*-acetyl glucosamine, amygdalin, arbutin, esculin ferric citrate, salicin, cellobiose, maltose, lactose, trehalose, gentiobiose, turanose, tagatose, and potassium gluconate. *L. paracasei* subsp. *paracasei* KCCM 40995, a type strain of *L*. *paracasei*, showed a utilization pattern similar to *L. paracasei* EPS DA-BACS. (Supplementary Table S1).

*Lacticaseibacillus paracasei* has health-promoting properties, including antimicrobial and antibiofilm, immune system stimulating, and anti-inflammatory, antioxidant, anti-obesity, and anti-proliferative/proapoptotic activities [2].

### 3.2. Visualization of EPS

**Figure 1:**
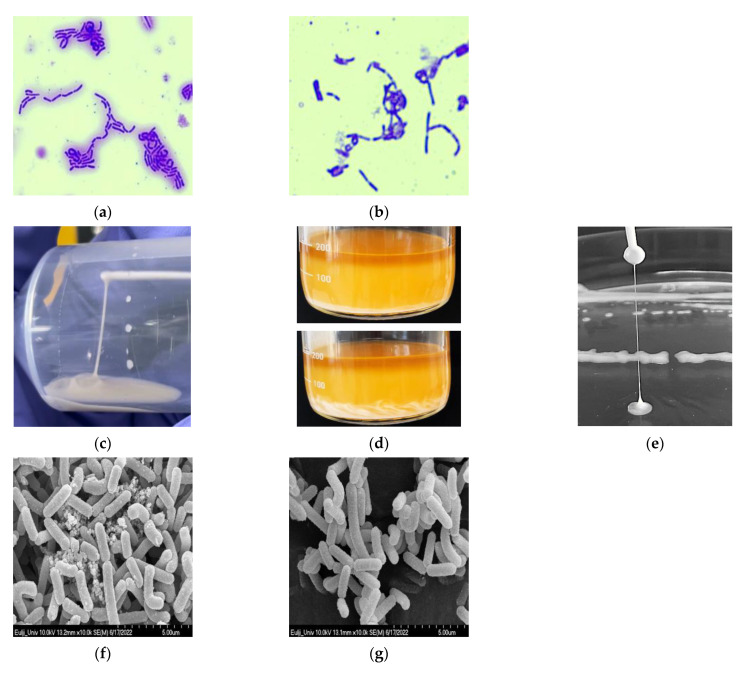
Visualization of EPS produced by *L. paracasei* EPS DA-BACS. (**a**) Crystal violet staining of *L. paracasei* EPS DA-BACS culture; (**b**) Crystal violet staining of *L. paracasei* EPS DA-BACS culture after centrifugation; (**c**) Ropiness of *L. paracasei* EPS DA-BACS’s culture broth; (**d**) Separation of *L. paracasei* EPS DA-BACS’s culture broth layer; (**e**) Ropiness of *L. paracasei* EPS DA-BACS’s colony; (**f**) Scanning electron microscopy image of *L. paracasei* EPS DA-BACS culture without removal of EPS; (**g**) Scanning electron microscopy image of *L. paracasei* EPS DA-BACS cells washed with PBS three times.

Because the selected *L. paracasei* EPS DA-BACS strain produced high amounts of EPS, the EPS produced by the strain was visualized by staining with crystal violet. When the *L. paracasei* EPS DA-BACS culture was stained with crystal violet, the cells and EPS around the cells were stained purple. When the cells producing EPS were separated by centrifugation and stained, only the cells stained purple (Figure 1a,b). This result showed that EPS produced by cells could be easily removed by centrifugation.

Many exopolysaccharides are highly charged, which aids the absorption of water and ions [25]. Exopolysaccharides are mainly composed of carbohydrate fractions, including L-fucose, D-glucose, D-mannose, D-galactose, L-rhamnose, D-galacturonic acid, D-glucuronic acid, L-guluronic acid, D-mannuronic acid, *N*-acetyl-D-glucosamine, *N*-acetyl-D-galactosamine, and non-carbohydrate components that are rich in carboxyl, phosphate, sulfate, and pyruvate fractions. These functional groups increase the negative charge on exopolysaccharides and subsequently enhance their lipophilicity and interactions with metal ions and polysaccharides [25]. There are two types of EPS structures. One is homopolysaccharides or HoPs, which consist of only one type of two or more monosaccharides, including glucose, galactose, fructose, or rhamnose, and even contains acetylations, pyruvylations, and phosphorylations [26]. The major monosaccharide and the other is a heteropolysaccharide, or HePS, which consists of three to eight units containing EPSs produced by LAB are heteropolysaccharides. Heps are synthesized using intracellular enzymes, glycosyltransferases, and sugar nucleotides as substrates [27].

The ropiness of the colony of *L. paracasei* EPS DA-BACS was identified using an inoculating loop, and its length was measured to be 44 mm (Figure 1c). When ropy strains are used to ferment milk products, they provide smoother consistency and higher viscosity than non-ropy strains [28]. In a rat model, ropy EPS had lower serum cholesterol and a higher HDL/total cholesterol ratio than non-ropy EPS [29]. Ropy EPS produced by *Pediococcus parvulus* IOEB has been shown to be resistant to lysozyme treatment [30].

When EPS produced by *L. paracasei* EPS DA-BACS was identified using a scanning electron microscope, lumps were observed around the cells (Figure 1d). When *L. paracasei* EPS DA-BACS culture was washed with PBS, EPS was washed out of the cells (Figure 1e). EPS produced by *L. paracasei* IJH-SONE68 was not removed by washing with PBS and adhered to the cell surface [31]. Saravanan and Shetty [32] reported that the smooth surface of EPS is a desirable property for the production of plasticized biofilms, and the consistent polymeric matrix of biofilms provide mechanical stability.

LAB can produce EPS tightly associated with the cell surface by covalent bonds forming capsular polysaccharides and can also produce polysaccharides loosely bound to the cell by non-covalent interactions or produce polysaccharides released into the environment [33]. It was confirmed that *L. paracasei* EPS DA-BACS was surrounded by EPS, which was identified by crystal violet staining, and EPS produced by *L. paracasei* EPS DA-BACS was removed by centrifugation, as confirmed by scanning electron microscopy. This indicated that EPS produced by *L. paracasei* EPS DA-BACS was loosely bound to the cell surface and could be easily removed.

### 3.3. Proposed Structures of EPS from the L. paracasei EPS DA-BACS

**Figure 2:**
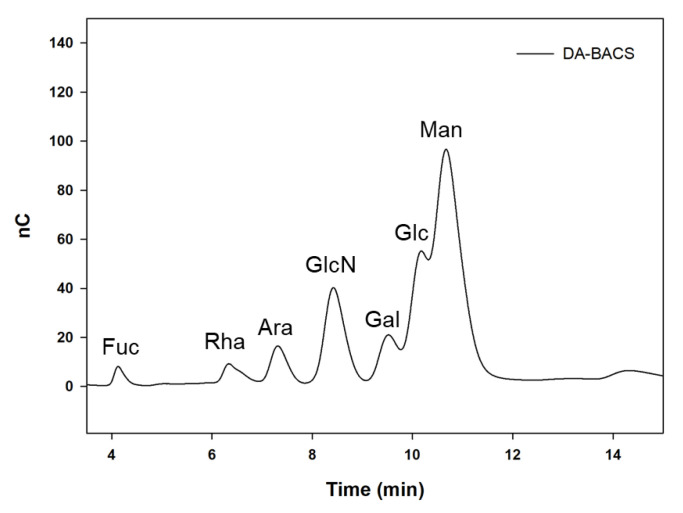
Monosaccharide compositions of EPS produced by *L. paracasei* EPS DA-BACS. Fuc, fucose; Rha, rhamnose; Ara, arabinose; GlcN, glucosamine; Gal, galactose; Glc, glucose; Man, mannose.

The monosaccharide composition of EPS produced by the *L. paracasei* EPS DA-BACS analyzed by HPAEC showed that glucose and mannose were major components in the structure (Figure 2), and other sugars (fucose, rhamnose, arabinose, glucosamine, and galactose) were also included. The molar content of mannose was higher than that of glucose, with a molar ratio of glucose:mannose of 0.46:1. Therefore, it was suggested that EPS from *L. paracasei* EPS DA-BACS was a glucomannan-type heteropolysaccharide.

Previous research has reported that the general structures of EPS from *Lactobacillus* species are glucan, mannan, and glucomannan-type EPS, depending on the microbial source [34]. The molar ratio of EPS from *L. paracasei* EPS DA-BACS differs from that of other glucomannan-type EPS (0.78:1) from *Lactiplantiobacillus plantarum* MTCC 9510 [35]. Additionally, EPS from *L. paracasei* EPS DA-BACS had a different minor sugar composition as glucosamine in its structure compared to a previous study [35]. Therefore, this result suggests that *L. paracasei* EPS DA-BACS produced glucomannan-type EPS with its own structural characteristics in terms of monosaccharide composition and molar ratio, compared to EPS from other *Lactobacillus* species.

**Figure 3:**
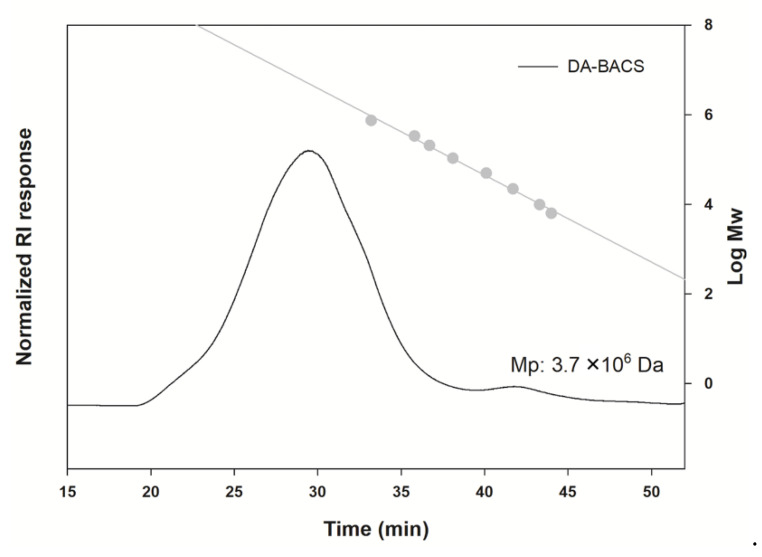
Molecular size distributions of EPS from *L. paracasei* EPS DA-BACS. Grey circles indicate pullulan standards.

The molecular sizes of EPS from *L. paracasei* EPS DA-BACS by relative comparison with pullulan standards are shown in Figure 3. EPS from *L. paracasei* EPS DA-BACS showed widely distributed molecular size ranges, and the peak molecular weight (Mp) of glycan was around 3.7 × 10^6^ Da according to the calibration curve. The broadly distributed molecular size of EPS from *L. paracasei* EPS DA-BACS is supported by previous research that glucomannan-type EPS from *Lactiplantiobacillus plantarum* BR2 showed a similar molecular distributed pattern (1.5–5.0 × 10^6^ Da; Mp: 2.4 × 10^6^ Da) [36].

**Figure 4:**
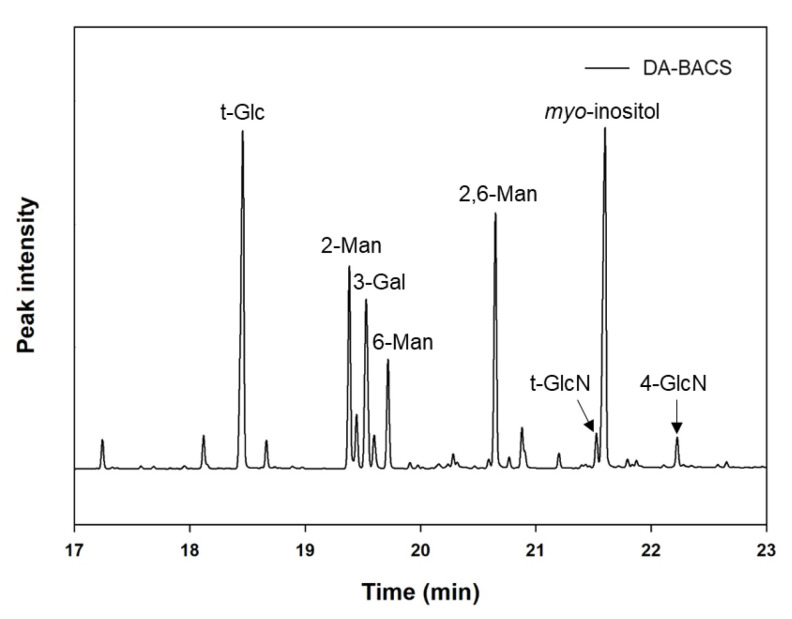
Major glycosyl types of EPS from *L. paracasei* EPS DA-BACS identified by mass spectra of PMAAs. *Myo*-inositol was the internal standard.

**Figure 5:**
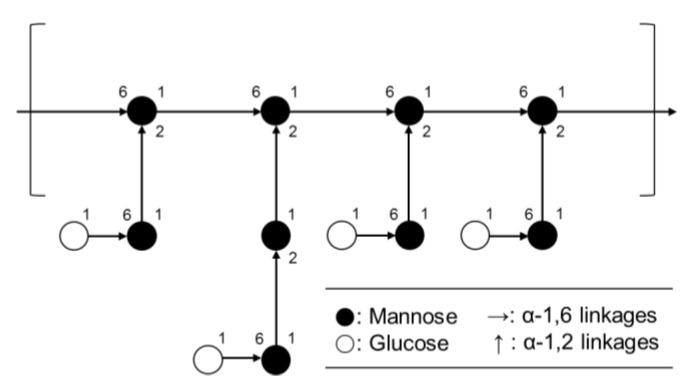
Schematic representation of the proposed structure of EPS from *L. paracasei* EPS DA-BACS.

EPS from *L. paracasei* EPS DA-BACS mainly consisted of mannosyl residues with 2-and 6-linear mannosyl chains and 2,6-branching points, according to the methylation analysis shown in Figure 4. This corresponds to the previously described monosaccharide composition results (Figure 2). Based on a previous study [37], this result suggested that EPS from *L. paracasei* EPS DA-BACS had main chains of a linear 1,6-mannose backbone and linear oligo-2-mannosyl chains with high branching degrees on the backbone. Glucosyl residues were linked at the end of the oligo-2-mannosyl chains. Galactose and glucosamine residues are expected to be linked to the mannose backbone or branch [38]. Figure 5 shows the proposed structure of EPS from *L. paracasei* EPS DA-BACS based on the structural analysis results and previous research.

Glucomannans consist of β-1,4 glycosidic bonds between D-glucose and D-mannose, with branches, acetylated hydroxyl groups, and hydroxyl functional groups on the chain. Li et al. reported that the degree of acetyl substitution and the mannan/glucose ratio was related to the expression of NF-κB, c-Jun, and phospho-c-Jun, which are pro-inflammatory signaling pathway proteins [38]. Molecular weight is related to mRNA expression, which in turn is related to immune products and nuclear transcription factor phosphorylation. It has been suggested that the structure of glucomannans correlates with macrophage activation performance [39].

Glucomannans are involved in the regulation of the immune system [40,41]. This regulation is divided into two groups: positive and negative regulation, which balance the immune system [42,43]. Glucomannans selectively promoted the secretion of inflammatory cytokines and increased the production of NO and reactive oxygen species. However, they have little effect on anti-inflammatory cytokine secretion. They also increase the viability of macrophages, suggesting that glucomannans have the potential to activate macrophages [39]. Because glucomannans are water-soluble, non-ionic hydrocolloidal dietary fibers, they have health-promoting effects, such as anti-diabetic, anti-obesity, laxative, prebiotic, and anti-inflammatory activities [44]. EPS produced by *L. paracasei* EPS DA-BACS are suggested to also have prebiotic and anti-inflammatory effects.

### 3.4. Tolerance to Gastrointestinal Environment

As shown in Table 1, *L. paracasei* EPS DA-BACS cells showed high tolerance to acids, bile salts, and pancreatin. *L. paracasei* EPS DA-BACS cells in the culture broth, in which the cells were surrounded by EPS, showed a higher acid tolerance than EPS-free cells. This result indicated that EPS produced by *L. paracasei* cells could impart acid tolerance to the cells and promote the survival rate of the cells in gastrointestinal conditions. The bile salt tolerance and pancreatin tolerance of EPS-containing *L. paracasei* EPS DA-BACS cells and EPS-free *L. paracasei* EPS DA-BACS cells were not statistically different. These results suggest that EPS produced by *L. paracasei* EPS DA-BACS promotes tolerance to gastrointestinal stress.

Mulaw et al. [45] reported that lactobacilli isolated from fermented Ethiopian food products showed 90.28–97.11% survival rates at pH 3 for 3 h. Sornsenee et al. [46] showed that when *L. paracasei* T0601, T0602, T0603, T0901, T0902, T1301, T1304, and T1901 strains were examined, the survival rates at pH 3, 0.3% bile salts, and 1 g/L pancreatin were 83.60–98.28%, 88.91–96.55%, 37.37–50.67%, respectively. Another report showed that when three lactobacilli strains from kefir samples in Malaysia were examined, the survival rates at pH 3 and 0.3% bile salts were 98.0 ± 3.3% and 96.89 ± 0.02%, respectively [47]. These reports indicated that *L. paracasei* EPS DA-BACS had a higher survival rate under gastrointestinal stress than *L. paracasei* studied by other researchers.

*L. paracasei* NFBC 338, which produces β-glucan, showed higher gastrointestinal and technological stress tolerance than non-β-glucan-producing bacteria [48]. The FAO/WHO guidelines established the criteria for probiotics in 2002 [1]. The criteria include survival under the gastrointestinal tract (GIT) condition (resistance to gastric acidity, bile acid resistance, pancreatin resistance), adherence to mucus and/or human epithelial cells and cell lines, and antimicrobial activity against potential pathogens. As the pH drops below 2, viability often decreases significantly; thus, the ability to survive under GIT conditions is essential [49,50].

However, the physiological role of EPS in LAB is not completely understood. Nevertheless, EPS plays an important role in protecting microbes against severe conditions, such as toxic metals, phage attacks, innate immune factors, and osmotic stress [7,51]. Additionally, the EPS layer could protect LAB from low pH, bile salts, and gastric and pancreatic enzymes, allowing LAB to survive in the gastrointestinal tract [7]. Moreover, *L. paracasei* CIDCA 8339, CIDCA 83123, and CIDCA 83124, in which the attached EPSs were removed by washing with PBS, showed a decrease in tolerance to stress conditions [52].

### 3.5. Gut Adhesion Ability Assay

The gut adhesion abilities of the culture broth of *L. paracasei* EPS DA-BACS and EPS-free *L. paracasei* EPS DA-BACS cells were similar, with values of 74.9% and 75.5%, respectively (Table 2). This indicates that EPS produced by *L. paracasei* EPS DA-BACS did not affect the adhesion properties of the cells. The gut adhesion ability of *L. paracasei* sup. *paracasei* BGSJ2-83 and BGHN14 were lower than 15% [53]. Fonseca et al. [54] reported that *L. paracasei* CCMA 0504 and CCMA 0505 showed gut adhesion values lower than 6%. Other studies have reported that *L. paracasei* sup. *paracasei* LC-01 showed 2% gut adhesion ability, and 19 other *L. paracasei* strains showed 65% gut adhesion ability [55,56].

Several studies have suggested that EPS may promote adhesion or reduce adhesion by masking cell wall adhesins [57]. *L. paracasei* DG showed no significant effect on the adhesion of Caco-2 cells [58]. It has been reported that the intestinal mucosa is directly contacted by EPS from LAB, which exert strain-dependent effects on adhesion [59,60]. Probiotics, which are usually administered orally, must survive in the gastrointestinal condition to reach and colonize the gut. The ability of a probiotic strain to produce EPS can be a significant advantage in demonstrating its health effects in situ [2].

### 3.6. Antibacterial and Antifungal Activity of LAB

**Figure 6:**
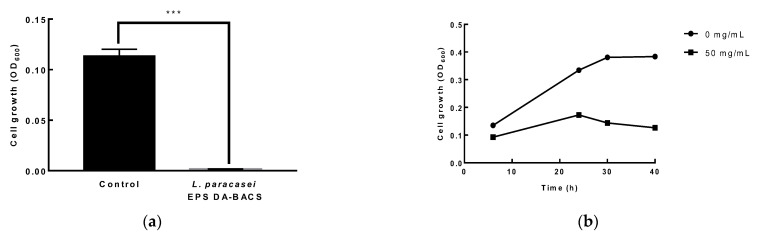
Growth inhibitory activity of *L. paracasei* EPS DA-BACS against *C. difficile*. (**a**) Culture supernatant of *L. paracasei* EPS DA-BACS was added to *C. difficile* culture; (**b**) 50 mg/mL of purified EPS was added to *C. difficile* culture. Asterisks show significant differences among groups at ***, *p* < 0.001 (*n* ≥ 3) using one-way ANOVA and Dunnett’s test.

When the culture broth of *L. paracasei* EPS DA-BACS was added to *C. difficile* culture, the growth of *C. difficile* was completely inhibited (Figure 6a). When 50 mg/mL of purified EPS produced by *L. paracasei* EPS DA-BACS was added to *C. difficile* culture, the growth of *C. difficile* decreased by 67% after 40 h of incubation (Figure 6b). This result showed that purified EPS itself had growth inhibitory activity against *C. difficile*, and *L. paracasei* EPS DA-BACS cells or its metabolites also showed growth inhibitory activity.

*C. difficile* is a toxin-producing bacterium that infects humans via the fecal-oral route. Its infection is spread by spores that are resistant to heat, acids, and antibiotics [61]. *C. difficile* produces two pathogenic types, enterotoxin A and enterotoxin B. It colonizes the gut, causes antibiotic-associated diarrhea, and is the major etiologic agent of pseudomembranous colitis [62]. Schoster et al. [63] suggested that probiotic strains use their acidic products to inhibit the growth of C. difficile in a pH-dependent manner.

The antimicrobial activity of EPS can be explained in vivo as follows: the prebiotic effect that helps LAB in gut colonization, the protection of commensal microorganisms from the adaptive immune response in the host, the enhancement of their competition with pathogenic bacteria, the inhibition of the growth of pathogenic microorganisms, the interference with their adhesion to the intestinal epithelium, and the prevention or reduction of the formation of biofilms of pathogenic bacteria [27]. Lactobacilli can produce bacteriocins that help protect the host from a wide range of pathogens, such as *C. difficile* [64] and *Vibrio parahaemolyticus* [65]. A common side effect of antibiotic therapy in hospitalized patients is *C. difficile* infection and depends on whether the lactobacilli strain has antagonistic activity against *C. difficile* in vitro [66].

**Figure 7:**
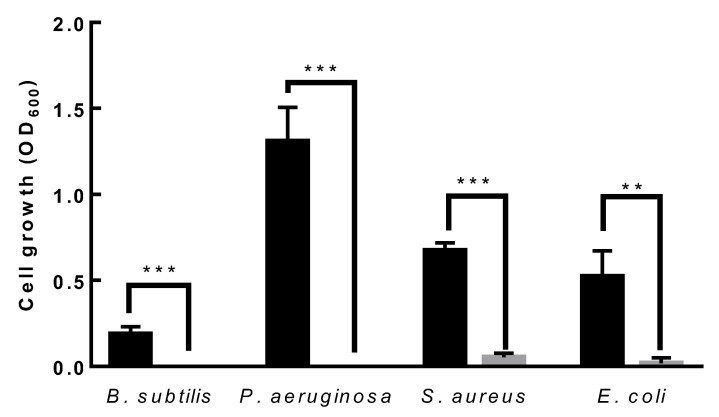
Antibacterial activity of *L. paracasei* EPS DA-BACS. The culture broth of indicator strains was inoculated in a 96-well plate at the final concentration of 1%(*v*/*v*) and incubated at 37 °C for 24 h. Black bar, the culture of indicator strains grown in MH medium; gray bar; *L. paracasei* EPS DA-BACS was added to the culture of indicator strains. Asterisks show significant differences among groups at **, *p* < 0.01; ***, *p* < 0.001 (*n* ≥ 3) using one-way ANOVA and Dunnett’s test.

When the antibacterial activities of *L. paracasei* EPS DA-BACS and the 12 other strains were examined, only *L. paracasei* EPS DA-BACS inhibited the growth of all indicator strains: *E. coli*, *Bacillus subtilis*, *P. aeruginosa*, and *S. aureus*. In particular, the 12 LAB strains, except *L. paracasei* EPS DA-BACS, did not inhibit the growth of *S. aureus* (Supplementary Table S2). *L. paracasei* EPS DA-BACS completely inhibited the growth of *Bacillus subtilis* and *P. aeruginosa* (Figure 7).

A previous report showed that *L. paracasei* M7 exhibited antibiofilm activity against bacterial pathogens such as *P. aeruginosa*, *Enterococcus faecalis*, *Bacillus subtilis*, *Bacillus cereus*, and *S. aureus* [14].

**Figure 8:**
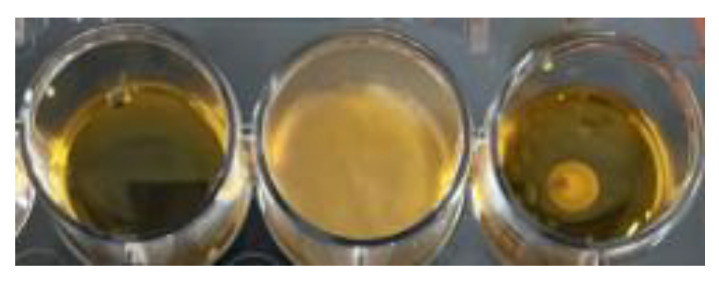
Antifungal activity of *L. paracasei* EPS DA-BACS. Left well, yeast malt soft agar without inoculation as the blank; middle well, yeast malt soft agar inoculated with *A. brasiliensis* followed by incubation at 25 °C for 48 h; right well, *L. paracasei* EPS DA-BACS culture was inoculated at the center of a well and incubated for 24 h at 37 °C, and *A. brasiliensis* was inoculated and incubated at 25 °C for 48 h.

Assessment of the antifungal activity using *A. brasiliensis* as an indicator strain revealed that *L. paracasei* EPS DA-BACS completely inhibited the growth of *A. brasiliensis* as shown in Figure 8. The EPS layer may play an important role in decreasing fungal hyphal formation [67]. In the in vitro gut model, EPS produced by *L. rhamnosus* GG decreased hyphal formation and reduced the adhesion of *Candida* sp. by co-aggregation, immunomodulation of the host epithelium, and competition for binding sites [68].

### 3.7. Anti-Inflammatory Activity of L. paracasei EPS DA-BACS

**Figure 9:**
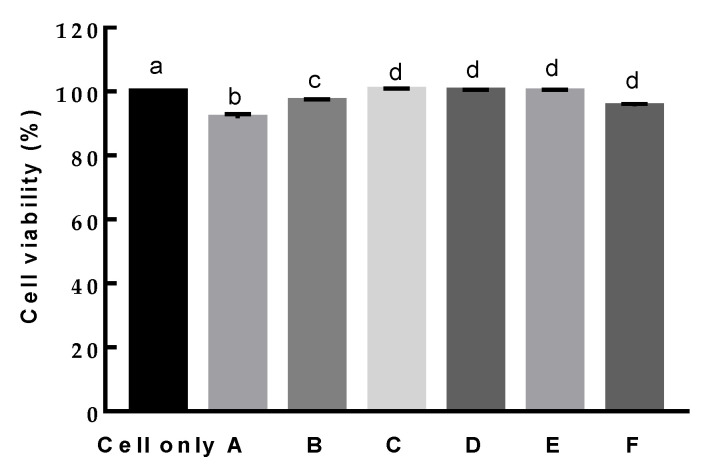
Viability of RAW 264.7 cells after treatment with *L. paracasei* EPS DA-BACS. A, 1.0 ×10^9^ CFU/mL; B, 5.0 × 10^8^ CFU/mL; C, 1.0 × 10^8^ CFU/mL; D, 5.0 × 10^7^ CFU/mL; E, 1.0 × 10^7^ CFU/mL; F, 1.0 × 10^6^ CFU/mL. Different letters show significant differences among groups at *p* < 0.05 (*n* ≥ 3) using one-way ANOVA and Duncan’s test.

When the cytotoxicity of *L. paracasei* EPS DA-BACS in RAW 264.7 cells was examined, *L. paracasei* EPS DA-BACS showed no cytotoxicity in all treated groups (Figure 9).

**Figure 10:**
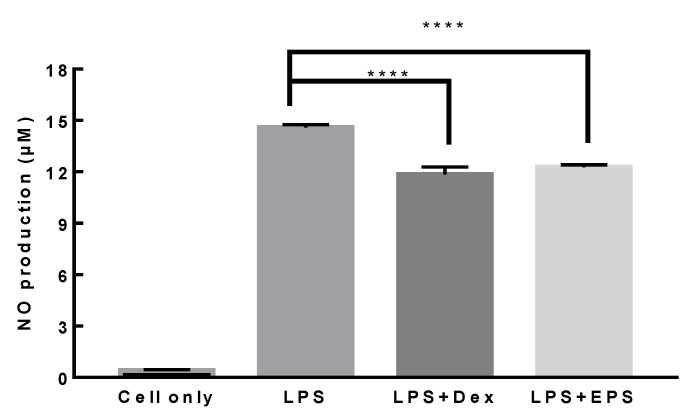
Anti-inflammatory activity of *L. paracasei* EPS DA-BACS. LPS, 1 μg/mL of LPS was added to RAW 264.7 cells and incubated for 24 h; LPS+Dex, 1 µM of dexamethasone was added to RAW 264.7 cells and incubated for 2 h, and 1 μg/mL of LPS was added and incubated for 24 h; LPS+EPS, 1.0 × 10^9^ CFU/mL of heat-treated *L. paracasei* EPS DA-BACS was added to RAW 264.7 cells and incubated for 2 h, and 1 μg/mL of LPS was added and incubated for 24 h. **** show significant differences among groups at *p* < 0.0001 (*n* ≥ 3) using one-way ANOVA and Dunnett’s test.

Because some LAB exhibit anti-inflammatory activity on macrophages, the anti-inflammatory activity of heat-treated *L. paracasei* EPS DA-BACS on RAW 264.7 macrophage cells was examined. When 1 μg/mL LPS was added, the concentration of NO produced was 14.6 μM. When 1 μM dexamethasone, a representative chemical that relieves inflammation, was added to RAW 264.7 cells, NO production was significantly decreased. Similar to dexamethasone treatment, treatment with heat-treated *L. paracasei* EPS DA-BACS also decreased NO production (Figure 10). These results showed that heat-treated *L. paracasei* EPS DA-BACS had considerable anti-inflammatory activity.

NO is a free radical produced by L-arginine by NO synthase reaction [69]. Macrophage activation promotes immune response by increasing the production of NO and cytokines [70]. Li et al. [71] reported that heat-killed *L. rhamnosus* GG diminished LPS-induced pro-inflammatory mediators and increased anti-inflammatory mediators in the liver, lungs, and plasma. The treatment of macrophages with heat-killed *Lactiplantibacillus plantarum* L-137 produces IL-12 [72].

### 3.8. Prebiotic Activity

**Figure 11:**
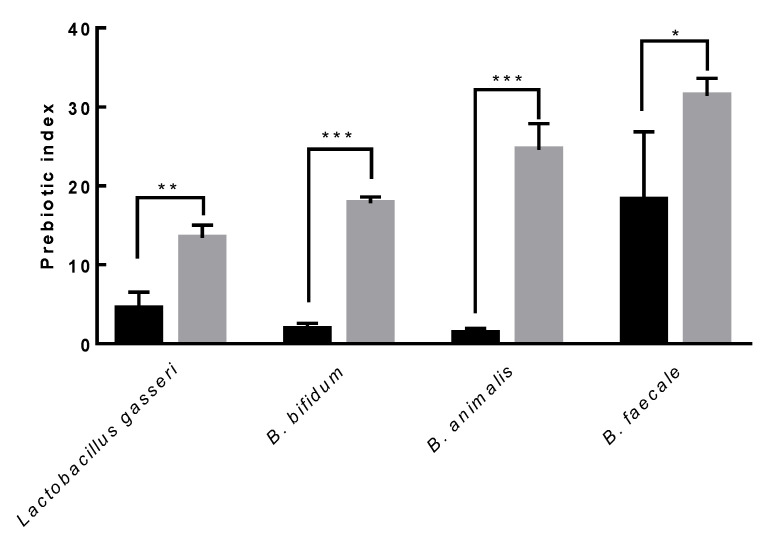
Prebiotic effect of EPS from *L. paracasei* EPS DA-BACS on probiotic strains. Black bar is inulin and gray bar is EPS from *L. paracasei* EPS DA-BACS. Asterisks show significant differences among groups at *, *p* < 0.05; **, *p* < 0.01; ***, *p* < 0.001 (*n* ≥ 3) using one-way ANOVA and Dunnett’s test.

The prebiotic index of purified EPS produced by *L. paracasei* EPS-DA BACS was evaluated to examine the prebiotic activity of representative probiotic strains, such as *Lactobacillus gasseri*, *B. bifidum*, *B. animalis*, and *B. faecale.* The prebiotic index of purified EPS produced by *L. paracasei* EPS-DA BACS was higher than that of inulin, a representative prebiotic carbohydrate, in all the probiotic strains used in this study (Figure 11). The prebiotic index of purified EPS produced by *L. paracasei* EPS-DA BACS was highest when treated with *B. faecale.* This result indicated that EPS produced by *L. paracasei* EPS-DA BACS had higher prebiotic activity than inulin and could be used as a bifidogenic factor. EPS produced by *L. paracasei* EPS DA-BACS promoted the growth of probiotic strains but not of pathogens such as *E. coli*, *Bacteroides fragilis*, and *C. difficile*.

Prebiotics are defined as substrates that are selectively utilized by host microorganisms conferring health benefits [73]. LAB produce EPS in complex structures. Therefore, EPS can resist gastric and intestinal degradation that reaches the intestinal level by acting as a substrate for bacteria in the intestinal tract. At the intestinal level, EPS acts as a prebiotic that stimulates the growth of beneficial bacteria and the production of metabolites such as short-chain fatty acids (SCFA), which play key roles in host health [7,51]. Prebiotics promote the growth of *Lactobacillus* and *Bifidobacterium* genera with probiotic properties. *L. paracasei* subsp. *paracasei* LB-8 EPS can regulate intestinal bacteria by growing *B. breve* at levels comparable to those enabled by inulin, a well-known prebiotic [74]. However, other reports have shown that the targets of prebiotics were expanded to include the promotion of other genera, such as *Eubacterium*, *Propionibacterium*, *Roseburia*, and *Akkermansia*, which can produce SCFA [73,75].

### 3.9. Analysis of the Whole Genome Sequencing of L. paracasei EPS DA-BACS

Whole genome sequencing was performed to identify antibiotic resistance (AMR) genes and virulence genes in the genome of *L. paracasei* EPS DA-BACS. Analysis of the basic features of the whole genome sequence revealed that the whole genome sequence of *L. paracasei* EPS DA-BACS consisted of 63 contigs with a genome size of 3,023,873 bp. The G+C content was 46.3%, with 2942 coding sequences, eight rRNA genes, and 55 tRNA genes.

When antibiotic resistance genes were analyzed using the RGI program, no perfect hits, one strict hit, or 205 loose hits were found. By filtering <70% identity hits for matching CARD references, the AMR gene was obtained with three loose hits for elfamycin, rifamycin, and fusidic acid, and one strict hit for disinfecting agents and antiseptics (Table 3). A matching AMR gene was not identified by ResFinder analysis. No virulence genes were found in the *L. paracasei* DA-BACS genome when the database of virulence genes from four pathogens (*Enterococcus*, *Escherichia coli*, *Listeria*, and *Staphylococcus aureus*) was used.

Although elfamycin, rifamycin, and fusidic acid were detected as loose hits, these antibiotics were not included in the list of important human antibiotics in the ESFA guidelines [24]. There was one strict hit for disinfecting agents and antiseptics; however, the % identity was too low to be considered. No perfect hits were found that detected a perfect match between the selected reference sequence and the mutation in the CARD.

### 3.10. Antibiotics Susceptibility

Analysis of the antibiotic susceptibility of *L. paracasei* EPS DA-BACS for nine antibiotics using the E-test revealed a MIC of all antibiotics below the cut-off value, indicating that the antibiotic susceptibility of *L. paracasei* EPS DA-BACS was acceptable (Table 4).

Probiotics must be safe for human consumption. Probiotic bacteria have the potential to confer resistance to a wide range of antibiotics [76,77]. Therefore, it is important to perform a minimum safety assessment to prevent clinical threats including antibiotic resistance. Typically, an antibiotic susceptibility test for vancomycin is not required because vancomycin resistance is often present in lactobacilli spontaneously, and these bacteria cannot transmit antibiotic resistance genes across isolates and species [78]. Similarly, Talib et al. [47] reported that all *Lactobacillus* isolates were resistant to vancomycin and gentamycin.

## 4. Conclusions

This study was designed to evaluate the probiotic properties of *L. paracasei* EPS DA-BACS, which produces exopolysaccharides. *L. paracasei* EPS DA-BACS showed high acid, bile, and pancreatin tolerance, and these properties increase the survival rate of cells in the gastrointestinal environment. Acid tolerance of cells is promoted in the presence of EPS, indicating that EPS has a protective function for cells to survive in the gastrointestinal tract. EPS also had growth-promoting activity on representative probiotic cells, such as *Lactobacillus gasseri*, *B. bifidum*, *B. animalis*, and *B. faecale.* In this study, we confirmed that EPS produced by *L. paracasei* EPS DA-BACS is glucomannan, the structure of which is different from that of other lactic acid bacteria. The result that EPS from *L. paracasei* EPS DA-BACS had growth inhibitory activity against *C. difficile* showed that EPS could be used for the treatment of *C. difficile*-related diseases such as Crohn’s disease. EPS from *L. paracasei* EPS DA-BACS also had higher growth promotion activity on probiotic strains than inulin, which showed that EPS can be developed as a prebiotic material. The culture broth of *L. paracasei* EPS DA-BACS containing EPS showed high antimicrobial activity against some pathogens as well as antifungal and anti-inflammatory activities. This study confirmed that *L. paracasei* EPS DA-BACS showed good probiotic properties, and EPS produced by this strain enhanced the tolerance to gastrointestinal stress and probiotic properties of the strain, suggesting the possibility of developing novel probiotic resources.

## Figures and Tables

**Table 1:** Gastrointestinal tract tolerance of *L. paracasei* EPS DA-BACS.

GIT Tolerance (%)	*L. paracasei* EPS DA-BACS	
	Culture Broth (In the Presence of EPS)	Pellet * (In the Absence of EPS)
Acid	101 ± 0.5 ^a^	98 ± 0.7 ^b^
Bile Salt	102 ± 5.3 ^a^	97 ± 0.5 ^a^
Pancreatin	101 ± 0.7 ^a^	99 ± 0.8 ^a^

The results are expressed as the means ± SD of three independent replicates. * Pellet, cells were washed three times with 0.85% (*w*/*v*) NaCl and resuspended in 5 mL basal MRS containing 2% (*w*/*v*) sucrose with 1000 units pepsin/mL, pH 3.0, for acid tolerance, with 0.3% (*w*/*v*) Bacto Oxgall for bile salt tolerance, and with 0.5% pancreatin for pancreatin tolerance. Different letters show significant differences among groups at *p* < 0.05 (*n* ≥ 3) using one-way ANOVA and Duncan’s test.

**Table 2:** Gut adhesion ability of *L. paracasei* EPS DA-BACS.

	*L. paracasei* EPS DA-BACS	
	Culture Broth (In the Presence of EPS)	Pellet * (In the Absence of EPS)
Gut Adhesion Ability (%)	74.9 ± 1.4 ^a^	75.5 ± 1.2 ^a^

The results are expressed as means ± SD of three independent replicates. * Pellet, cells were washed three times with DPBS and resuspended in 1 mL DMEM containing 10% fetal bovine serum. Different letters show significant differences among groups at *p* < 0.05 (*n* ≥ 3) using one-way ANOVA and Duncan’s test.

**Table 3:** Results of ARG analysis based on genome sequencing data.

RGI Criteria	AMR Gene Family	Drug Class	% Identity Matching Region
Strict	Small multidrug resistance (SMR) antibiotic efflux pump	Disinfecting agents and antiseptics	38.24
Loose	Elfamycin resistant EF-Tu	Elfamycin antibiotic	72.19
	Rifamycin-resistant beta-subunit of RNA polymerase (*rpoB*)	Rifamycin antibiotic	71.59
	Antibiotic resistant *fusA*	Fusidic acid	72.19

**Table 4:** Antibiotics susceptibility of *L. paracasei* EPS DA-BACS.

Antibiotics	Cut-Off Value (µg/mL) *	MIC	Susceptibility	Assessment
		(µg/mL)		
Ampicillin	4	0.25	S ***	Acceptable
Vancomycin	n.r. **	n.r.	n.r.	Acceptable
Gentamycin	32	4	S	Acceptable
Kanamycin	64	48	S	Acceptable
Streptomycin	64	24	S	Acceptable
Erythromycin	1	0.125	S	Acceptable
Clindamycin	1	0.023	S	Acceptable
Tetracycline	4	1	S	Acceptable
Chloramphenicol	4	4	S	Acceptable

* Cutoff value was established in the EFSA guidelines. ** n.r., not required. *** S, Susceptible.

## Data Availability

Not applicable.

## Supplementary Materials

The following supporting information can be downloaded at https://www.mdpi.com/article/10.3390/microorganisms10122431/s1, Figure S1: Phylogenetic tree of *Lacticaseibacillus paracasei* EPS DA-BACS generated by the neighbor-joining method based on the sequence of the 16S rRNA gene; Table S1: Carbohydrate utilization of *Lacticaseibacillus paracasei* EPS DA-BACS analyzed by API CHL 50 kit; Table S2: Antimicrobial activity of 12 *Lacticaseibacillus paracasei* strains.
