# Supplementary material for: Potential Probiotic Properties of Exopolysaccharide-Producing Lacticaseibacillus paracasei EPS DA-BACS and Prebiotic Activity of Its Exopolysaccharide
Source: Microorganisms. 2022 Dec 8;10(12):2431. doi: 10.3390/microorganisms10122431 (PMC9787920; doi:10.3390/microorganisms10122431)
Supplement: Supplementary file 1 [file microorganisms-10-02431-s001.zip › microorganisms-2082061-supplementary.pdf]

Supplementary data

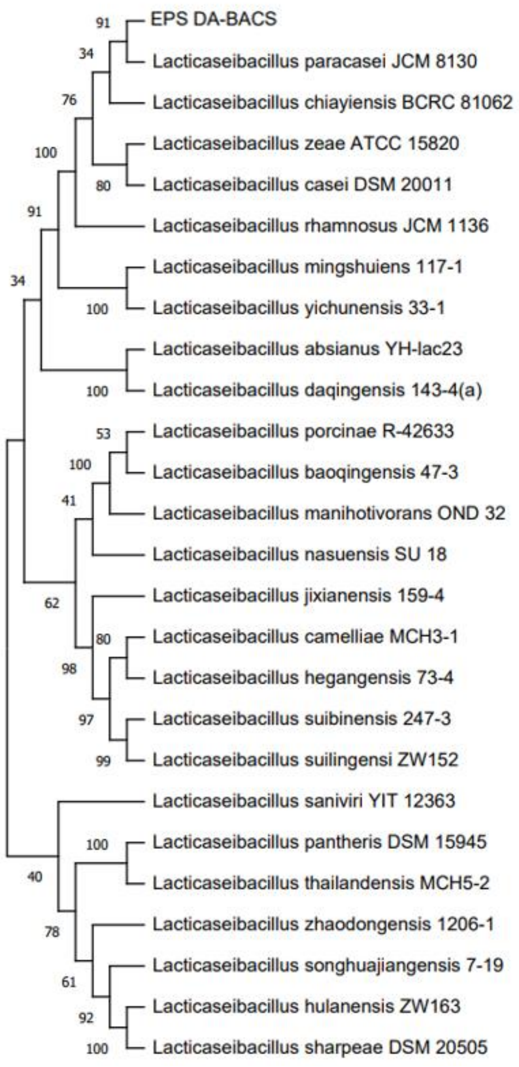

Figure S1. Phylogenetic tree of *Lacticaseibacillus paracasei* EPS DA-BACS generated by neighbor-joining method based on the sequence of 16S rRNA gene.

Table S1. Carbohydrate utilization of *Lacticaseibacillus paracasei* EPS DA-BACS analyzed by API CHL 50 kit.

Carbohydrate	strains		Carbohydrate	strains	
	EPS DA-BACS	KCCM 409915		EPS DA-BACS	KCCM 409915
Glycerol	-	-	Salicin	+	+
Erythritol	-	-	D-Cellobiose	+	+
D-Arabinose	-	-	D-Maltose	+	-
L-Arabinose	-	-	D-Lactose (bovine origin)	+	+
D-Ribose	+	+	D-Melibiose	-	-
D-Xylose	-	-	D-Saccharose (sucrose)	-	-
L-Xylose	-	-	D-Trehalose	+	+
D-Adonitol	-	-	Inulin	-	-
Methyl-β-D-Xylopyra-noside	-	-	D-Melezitose	-	+
D-Galactose	+	+	D-Raffinose	-	-
D-Glucose	+	+	Amidon (starch)	-	-
D-Fructose	+	+	Glycogen	-	-
D-Mannose	+	+	Xylitol	-	-
L-sorbose	+	-	Gentiobiose	+	+
L-Rhanmose	-	-	D-Turanose	+	+
Ducitol	-	-	D-Lyxose	-	-
Inositol	-	-	D-Tagatose	+	+
D-Mannitol	+	+	D-Fucose	-	-
D-Sorbitol	+	-	L-Fucose	-	-
Methyl-α-D-Mannopyra-noside	-	-	D-Arabitol	-	-
Methyl-α-D-Glucopyra-noside	-	-	L-Arabitol	-	-
N-Acetyl Glucosamine	+	+	Potassium Gluconate	+	+
Amygdalin	+	-	Potassium 2-Ketoglu-conate	-	-
Arbutin	+	-	Potassium 5-Ketoglu-conate	-	-
Esculin ferric citrate	+	+			

L. paracasei KCCM 40995 was used as a reference strain.

Table S2. Antimicrobial activity of twelve *Lacticaseibacillus paracasei* strains

	KCTC 3169	KCTC 13090	KCTC 3165	KCTC 3189	KCTC 5546	KCTC 3510	KCTC 5058	KCTC 3074	KCCM 40995	KCCM 42830	KCCM 32822	KCCM 41246
Bacillus subtillis	-	-	-	-	-	-	+	-	-	-	-	-
P. aeruginosa	-	-	-	-	-	-	+	+	-	-	-	-
S. aureus	+	+	+	+	+	+	+	+	+	+	+	+
E. coli	-	-	-	-	-	+	+	+	-	-	-	+

+, > 0.1 OD_{600}; -, < 0.1 OD_{600}
